# Finding common development paths in voluntary national reviews reporting on sustainable development goals using aspect-based sentiment analysis

**DOI:** 10.1371/journal.pone.0307886

**Published:** 2024-08-14

**Authors:** Christoph Funk, Elena Tönjes, Ramona Teuber, Lutz Breuer

**Affiliations:** 1 Center for International Development and Environmental Research (ZEU), Justus Liebig University, Giessen, Germany; 2 Department of Economics, Chair of Statistics and Econometrics, Justus Liebig University, Giessen, Germany; 3 Institute for Agricultural Policy and Market Research, Justus Liebig University Giessen, Giessen, Germany; 4 Institute for Landscape Ecology and Resources Management (ILR), Research Centre for Bio Systems, Land Use and Nutrition (iFZ), Justus Liebig University Giessen, Giessen, Germany; Shanghai Maritime University, CHINA

## Abstract

Voluntary National Reviews (VNRs) provide a platform for participating countries to share their experiences, failures, and successes in achieving the United Nations (UN) Sustainable Development Goals (SDGs). The objective of this study is to gain a deeper understanding of the narrative elements, particularly the sentiment, in VNRs in order to more effectively assess and support global SDG progress. A total of 232 VNRs from 166 countries are analyzed using Aspect-Based Sentiment Analysis (ABSA) to extract each country’s sentiment toward the 17 SDGs. The sentiment scores are then compared to the corresponding official UN SDG scores, and countries are grouped by their sentiment toward all 17 SDGs to identify potential common development pathways. The analysis uncovers a notable positive correlation between the reported sentiment and official SDG scores for SDG 2 (zero hunger) and SDG 11 (sustainable cities and communities), and a negative correlation for SDG 5 (gender equality). Conversely, this relationship is not significant for the majority of SDGs, suggesting that VNR narratives may not directly reflect actual progress. A t-distributed stochastic neighbor embedding (t-SNE) approach indicates a consistent sentiment score among developed countries. In contrast, there are greater differences in reporting sentiment among Emerging Markets, Frontier Markets, and Least Developed Countries (LDCs), where there is greater dispersion (especially among LDCs) and sentiment in reporting on SDG progress that appears to have changed from one reporting year to another. These findings highlight the need to interpret VNRs in the context of each country’s unique situation and challenges specific to each country.

## 1 Introduction

The United Nations’ (UN) 2030 Agenda, which outlines 17 Sustainable Development Goals (SDGs) and 169 targets, provides a structured framework for its member states to collectively achieve a better future. Voluntary National Reviews (VNRs) serve as a unique platform, allowing member states to share experiences, both successes and setbacks, as they navigate their paths towards achieving the 17 SDGs [1]. Consequently, VNRs provide textual insights into each country’s progress toward the SDGs. However, despite the structured framework and shared experiences, there is a growing concern that many countries may not fulfill the targets set for 2030, especially in light of the challenges caused by the COVID-19 pandemic [2].

This raises the question: How can VNRs be systematically evaluated to understand the progress of each nation towards the SDGs without manually reviewing the extensive VNR texts? Addressing this question is imperative, given the increasing evidence of delays and the need for transformative changes to redirect the world towards sustainable development [3, 4].

To address this gap, we employ Natural Language Processing (NLP) techniques to extract and analyze the textual data from VNRs. These methods are powerful for uncovering insights into each country’s SDG performance, identifying patterns, and understanding the interactions between different goals. The SDGs can have limited efficacy due to selective implementation that often overlooks their complex interactions. Such interactions, depending on specific contexts and locations, can manifest both as synergies, where one SDG positively influences another, or as trade-offs, where progress in one area may come at the expense of another [2]. This may extend beyond the interactions between SDGs to encompass those between countries. The existence of shared challenges or achievements may provide an opportunity for countries to collaborate and accelerate the achievement of SDGs. Consequently, the concept of synergies and trade-offs is not limited to the relationship between SDGs, but also extends to that between countries. In the event that countries encounter analogous challenges or attainments, they are said to share a common developmental paths. In this context, we proceed with our analysis, which involves examining potential synergies and trade-offs between countries, rather than between SDGs, when addressing them in our analysis. We aim to uncover trends in the way countries report on individual SDGs, shedding light on shared developmental paths on a global level.

While the existing literature on the topic of identifying common development paths is limited [5, 6], a number of studies have already revealed the dynamics between the SDGs and identified trade-offs and synergies. [7–12]. Network analysis techniques were employed by Le Blanc [7] to identify that some SDGs are interlinked with numerous other SDGs through multiple targets, whereas other SDGs exhibit a comparatively weaker connection to the wider system. Hegre et al. [8] employ a clustering algorithm, namely principal component analysis (PCA). This methodology enables the authors to identify trends, synergies, and trade-offs between SDGs. Their findings indicate the existence of synergies within and between SDGs, with the exception of SDG 10, in terms of both levels and temporal change. Pradhan et al. [9] also examine the interrelationships between the SDGs. In contrast with the previously discussed methodologies, the present approach involves the application of correlation analysis between SDG scores for over 200 countries. In addition, Kroll et al. [13], also rely primarily on the UN’s official SDG metrics to identify trends and patterns. They analyze how trade-offs and synergies have evolved globally in the recent past. Furthermore, Pham-Truffert et al. [10] seek to identify potential synergies and trade-offs between SDGs, employing a comprehensive literature review as the foundation for their analysis. Nilsson et al. [11] also address SDG interactions and trade-offs, although they adopt a somewhat more theoretical approach. The authors develop a new conceptual framework for understanding the interactions of SDGs, specifically by using case studies from the energy, health, and ocean sectors. This framework allows for the understanding of how interactions might differ between different factors, such as the time horizon or resource endowments. The authors posit that their framework has the potential to enhance scientific research and policy-making decisions by establishing a SDG Interactions Knowledge Platform, which would facilitate the exchange of knowledge about SDG interactions. In contrast to the papers previously mentioned, which examine direct synergistic or trade-off relationships between the SDGs, Xiao et al. [12] utilise methods such as a plus-minus decision-making trial and evaluation laboratory model in order to also examine the indirect effects of SDGs on each other. The results demonstrate that when indirect effects are considered, the synergy effect is predominant.

Research on SDGs encompasses not only the identification of synergies and trade-offs, but also the development of scores based on data that can be collected at the regional level. For example, D’Adamo et al. [14] utilize SDG scores provided by the Italian Institute of Statistics (ISTAT). The authors examine Italy in particular and find that the northern regions outperform the southern regions with regard to achieving the SDGs. Furthermore, Anselmi et al. [15] utilise numerical data in the form of the official SDG scores provided by the UN, with the intention of facilitating geographical comparisons. However, the comparison is conducted at the country level and is limited to European countries. The study also indicates that countries in the northern hemisphere, such as Sweden, Denmark, and the Netherlands, appear to excel in specific areas. Murphy et al. [4] also focus on the European Union in particular, developing a composite index where European countries are measured against the worst and best performing countries in terms of achieving the SDGs. In addition, the majority of extant literature employs the official SDG scores without raising any concerns regarding their quality [16, 17]. As the quality of the scores is open to question, we also examine whether the UN-provided SDG scores reflect the countries’ assessment on reaching the goals.

As the literature indicates, research on interactions and regional differences of SDGs is relatively extensive. However, there are clear gaps yet to be addressed. As the majority of existing literature focuses on the analysis of official SDG scores, there is a deficiency in research focusing on the systematic analysis of textual data on SDGs at the country level. Similar to our study, Sebestyén et al. [6] apply NLP techniques to VNRs. The authors primarily focus on network analyses, which is based on the co-occurrence of important keywords from the VNRs. Like this study, they attempt to cluster countries in order to gain insights into which countries may face similar challenges in achieving the SDGs. The authors employ a multiplex network analysis, where important keywords form central nodes in a network, and describe the significant fields of sustainable development. They then utilize the similarities between the networks to cluster countries. While numerical data can provide valuable insights, it is not always sufficient to fully capture the complete picture. Consequently, this may result in the overlooking of the nuances present in textual reports shared by the countries themselves. The purpose of our study is to address this research gap. A sentiment analysis of VNRs is being conducted with the objective of gaining a deeper understanding of how countries perceive their journey towards achieving the SDGs. By integrating the network analysis techniques of Sebestyén et al. [6] with our sentiment-based methodology, we present a novel text-based sentiment score for each SDG, offering a more comprehensive perspective than studies that rely solely on numerical data.

While the SDGs are crucial for global sustainability, evaluating how nations perceive their progress towards the SDGs remains a challenge. To gain insight into how countries perceive each SDGs progress in their VNRs, we employ a sophisticated sentiment analysis method, namely Aspect-Based Sentiment Analysis (ABSA). This allows us to generate a text-based SDG-related sentiment score for each country. To the best of our knowledge, this study is the first to apply fine-grained sentiment analysis to VNRs. In addition, given the significant financial demands of achieving the SDGs, especially for developing countries, and the critical role of capital markets, we further categorize countries based on their market strength and economic stage. We adopted the MSCI Inc. market classification framework [18] to categorize countries into Developed Markets (DM), Emerging Markets (EM), or Frontier Markets (FM), with Standalone Markets being subsumed under Frontier Markets. Additionally, we utilized the UN’s classification for Least Developed Countries (LDCs) [19]. This specific classification, while differing from traditional systems, was rooted in the updated M49 categorization [20]. All countries that do not fall into these classifications are referred to as ‘Others’. Our categorization approach also highlights the growing role of capital markets in achieving the SDGs [21, 22].

We aim to address the following research questions: 1. What countries are facing similar challenges and successes in their efforts to achieve the SDGs? 2. To what extent does the sentiment expressed in VNRs correlate with actual progress towards the SDGs? Specifically, does the sentiment align with or diverge from the countries’ perspectives on their progress? The remainder of this paper is organized as follows. Section 2 provides a comprehensive description of the data used. Section 3 presents the methodology to extract the text data and the ABSA model. Section 4 outlines the model validation, our main findings, common development paths and a comparison between sentiment score and actual country performance. A discussion of our results is provided in Section 5 followed by a conclusion in Section 6.

## 2 Data

The VNRs used in our study can be downloaded from the sustainable development platform (https://sustainabledevelopment.un.org/vnrs/) where 168 countries currently publish VNRs. We used all available reports between 2016 and 2021. The UN does not provide guidelines for the structure of VNRs; therefore, reports vary widely in length and layout [6]. The reports are published in PDF format only and they were extracted using the **VI**sual **LA**yout (VILA) layout parser described in Section 3.

**Fig 1:**
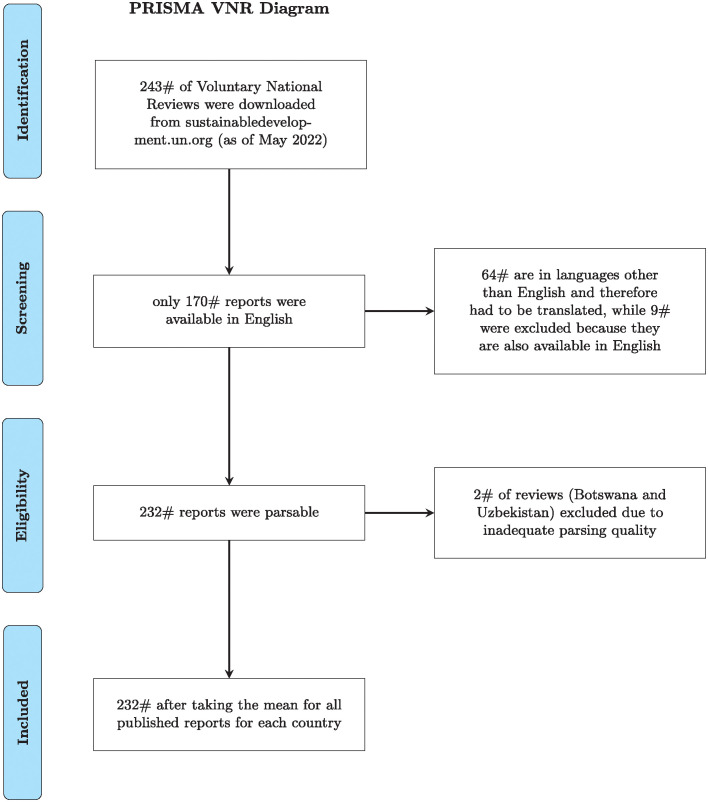
PRISMA flow diagram for dataset generation. This diagram outlines the systematic process employed for the creation of our final dataset, depicting the stages of selection, screening, eligibility, and inclusion. It provides a transparent and methodical overview of data curation specific to our research needs.

Fig 1 provides an overview of the steps involved in creating our dataset. After screening the available VNRs, we downloaded 243 VNRs published between 2016 and 2021. Out of these, 73 reports were not available in English, including a report from Bolivia which, despite being listed as English on the UN’s homepage, was written in Spanish. To integrate these into our dataset, we implemented the MarianMTModel and MarianTokenizer from the Helsinki-NLP group, which are part of the transformers library, to systematically translate sentences from French and Spanish into English. These non-English reports had to be translated for our analysis since our ABSA model operates exclusively on English-language text. Out of these 73 non-English reports, we did not use 9, because they were available in both English and another language (Spanish or French), and for consistency, we included only their English versions in our analysis. Of the remaining 170 PDFs that were parsed, reports from Botswana and Uzbekistan were excluded due to inadequate quality. In these cases, ‘insufficient quality’ refers to one report being a slideshow with minimal text, and the other being text in image form rather than in a machine-readable PDF format. Hence, we used 232 reports in our analysis. Table 1 provides an overview of the number of VNRs based on the MSCI Inc. market classification.

**Table 1:** Number of Voluntary National Reviews (VNRs) in our dataset based on country classification.

Country Classification	Number of VNRs
Developed Markets (DM)	29
Emerging Markets (EM)	40
Frontier-Markets (FM)	54
Least Developed Countries (LDC)	37
Others	72
Sum	232

An overview of all countries, their ISO3 code, length, and year of publication of our entire dataset can be found in S2 Table. The length of VNRs varies widely, ranging from an average of 28 pages over the years in Switzerland and the Maldives to 430 pages in Indonesia. The maximum number of pages for a single report in our sample is from Indonesia with 786 pages. Although most countries (110) have published one report so far, 46 have already published two reports, and ten countries have published three reports in the last 6 years. For each year we have the following numbers of reports available: 2016: 20; 2017: 41; 2018: 46; 2019: 44; 2020: 42; and 2021: 39 VNRs. For our analysis, we took the mean per country over all its published reports. As a result, we have 166 observations (i.e. countries) available for our analysis.

## 3 Methodology

### 3.1 From PDFs to raw text data

The VNRs are only available in PDF format, which requires text extraction prior to detailed analysis. Despite the popularity of Python modules like ‘PDFminer’ or ‘Py2PDF’, we found them inferior due to their inconsistent text quality extraction and omission of important layout details [23, 24]. In search of a more robust extraction tool, we employed a model originally intended for scientific articles, only to discover that it was also well-suited for VNRs. Developed by Shen et al. [24], the model uses VILA groups, it offers two model variants: the Visual Layout-guided Hierarchical Model (H-VILA) and the Injecting Visual Layout Indicators (I-VILA), both rooted in pre-trained language models like BERT [25]. The VILA model operates on the premise that text can be systematically segmented into lines or blocks, facilitating easier extraction using an OCR-backed, rule-based PDF parser.

For our study, we chose the H-VILA model pre-trained on the grotoap2 dataset [26], integrated with the layout-aware BERT model (layoutLM [23]), a combination that demonstrated superior extraction capabilities. Among the 232 VNRs, 14—presented in landscape mode—were split for effective extraction. The extraction yielded text classified by types: abstract, body content, and so on. Since our ABSA approach heavily relies on full sentences, we concentrated only on the main body content, deliberately excluding text from figures or tables. This method, though superior to its peers, was not foolproof and required additional text cleaning, elaborated in Table S1 Table. Furthermore, we modified certain source codes in the VILA site packages. To ensure text parsing even in the event of decoding errors, we implemented error-ignoring functionalities within the decoding functions of ‘PDFminer’ and ‘PDFplumber’.

### 3.2 Aspect-based sentiment analysis

To discern sentiment from the extracted VNR texts, we employed ABSA as proposed by Smith et al. [27]. This methodology, augmented with insights from various studies [28–32] and methodologies [33, 34], allowed us to pinpoint specific sentiments towards individual SDGs. Unlike vanilla sentiment analysis, ABSA is designed to capture a text’s sentiment toward a specific entity, such as a company, an individual, or a location [35].

An ABSA example introduced by Li et al. [36] is an end-to-end BERT layer model. Although powerful, this approach has its limitations; it relies on the explicit mention of the entity in the sentence. Given that VNRs often imply sentiments toward SDGs without naming them directly, we chose a sequence classification model [33, 34]. Rooted in the ‘SentiHood’ dataset introduced by Saeidi et al. [29], this model emphasizes targeted aspect-based analysis. In our application, we focused on the target sentiment analysis [37, 38], considering the SDG as the target and classifying the sentiment as positive, negative, or neutral. The code for the model we used and slightly modified can be found at https://github.com/mwbrulhardt/yelp-absa.

For our model’s training, we utilized all UN progress reports from 2016 to 2021. We partitioned 80% of the data for training and 20% for testing. To mitigate overfitting, we allocated 10% of the training data for validation [39–44]. Our ABSA required each sentence to be tagged with the appropriate SDG (from 1 to 17) and a sentiment. Given the structured nature of UN progress reports, divided by SDG progress, we effectively had a pre-tagged dataset. Manual reviews ensured accurate sentiment and SDG tags. For example, one section in the 2019 report covers SDG 1 and its progress. We reviewed each sentence by hand and tagged most of the sentences in this section with the appropriate entity ‘SDG1’. However, in a minority of cases, the sentences referred to a different goal and they were tagged accordingly. We (i.e. three people) then tagged the sentiment for each sentence and selected the majority sentiment for each of the resulting 2,079 sentences.

Addressing our dataset’s imbalance—specifically in the sentiments for SDGs 1, 2, 4–7, 11, and 16—was crucial. We tackled this by doubling the positive sentences, using the ContextualWordEmbsAug function from nlpaug and the ‘distilbert-base-uncased’ model [45]. This augmentation process increased our training dataset from 2,079 to 2,265 sentences.

In terms of model selection, we faced a range of choices. We settled on BERT-base uncased for a few reasons. While more extensive models might offer minor improvements, they come with longer training times. On the other hand, choosing a much smaller model could compromise the results. Leveraging the capabilities of an NVIDIA RTX A5000 GPU, we found that the BERT-base uncased struck the right balance between performance and efficiency.

We initially trained our model using the unbalanced dataset to evaluate the effectiveness of our data augmentation strategy. This comparison between the up-sampled model and the original was instrumental in validating our approach. To optimize the model’s performance, we conducted a systematic grid search on hyperparameters, focusing on ‘learning rate’, ‘batch size’, and ‘epochs’. The results indicated that the up-sampled model, when paired with the optimal hyperparameters, yielded superior performance over the model trained on the unbalanced dataset. Specifically, using a learning rate of 3e-5, a batch size of 16, and 15 epochs, the up-sampled dataset achieved an MCC of approximately 0.74489, whereas the non-augmented set secured an MCC of 0.70998.

**Fig 2:**
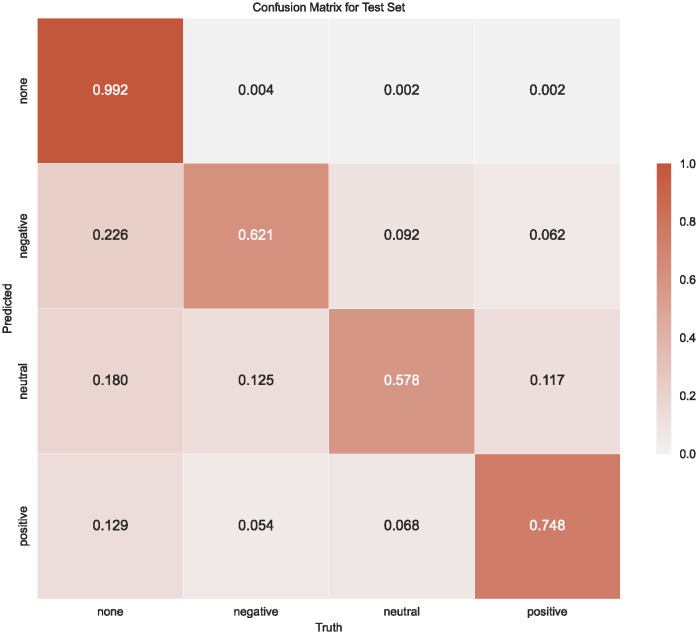
Confusion matrix for the training results of the ABSA model. This matrix contrasts the predicted sentiment labels (y-axis) against the true human-annotated labels (x-axis) across sentences. The sentiments are categorized into negative, positive, and neutral, with an additional ‘none’ category representing sentences where other sentiments not under discussion are present. A correct model prediction aligns a sentence with its respective SDG sentiment while categorizing all non-relevant SDGs as ‘none’.

Fig 2 shows the confusion matrix for the model with up-sampling. In contrast, without up-sampling, we only obtained lower values (true positive: 0.570; true negative: 0.602; true neutral: 0.525). The model with up-sampling predicts the minority class of positive sentiment better than the model without up-sampling does. The ‘none’ label is included in the matrix due to the specific output format the model generates. The model’s output for a sentence consists of one label for each SDG. The SDG, which the model predicts to be the entity discussed in the sentence, is assigned with the labels ‘positive,’ ‘negative,’ or ‘neutral.’ The other SDGs that the model does not predict to be discussed in the particular sentence are labeled ‘none’. Therefore, each sentence receives one polarity label for the SDG spoken about and 16 ‘none’ labels. The ‘none’ category has the highest value in the confusion matrix because there are far more ‘none’ labels in the test set. This is because each sentence has 16 ‘none’ labels attached and only one polarity label. Therefore, the model correctly predicted 99.2% of the ‘none’ labels, which can be interpreted as correctly predicting the specific entity addressed by the sentence.

The results produced by the model comprise entities, in this case one of the SDGs and the associated sentiment, which may be negative, positive, or neutral. Consequently, each sentence for each country and report is allocated to all SDGs, with either a “none” designation if the sentence does not address the SDG or one of the sentiments. In order to derive a sentiment score for our analysis, we assign numerical values to different sentiment labels. These values are as follows: a value of 1 for positive sentiment, a value of -1 for negative sentiment, and a value of 0 for neutral sentiment. Sentences labeled as ‘none’ are considered to be missing data. The sentiment score, which ranges between -1 and 1, is calculated by computing the mean sentiment score for each SDG based on all sentences within a single report per country. The mean is calculated by averaging the assigned numerical values, with missing data (NaNs) being excluded from the calculation. The resulting values represent the sentiment score that will be utilized for subsequent analysis.

**Fig 3:**
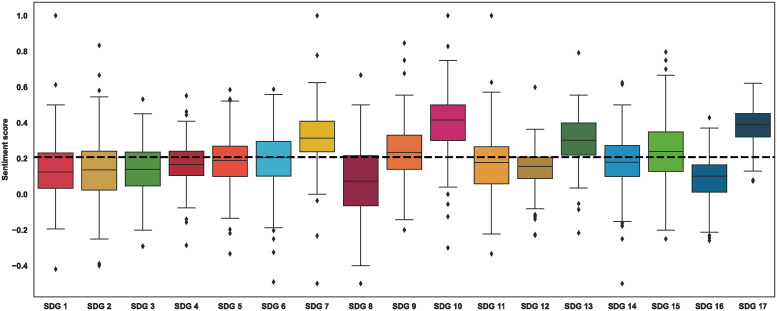
Boxplots of model-predicted sentiment scores for each SDG. This series of boxplots illustrates the distribution of the model-predicted sentiment scores for each of the 17 Sustainable Development Goals (SDGs). A black dashed line across the boxplots marks the overall mean of the median scores across all SDGs, which serves as a benchmark for comparison.

Fig 3 summarizes the sentiment score for each SDG in each country in our dataset. For the 56 countries that published more than one report, we took the average of the countries’ sentiment score, leaving us with 166 countries and their respective scores. We also calculated the median of these averages for comparison. We find that the median of the average sentiment scores is positive across all 17 SDGs at 0.209. However, we find that the sentiment score varies considerably across countries around the world. Medians of the mean sentiment score range from 0.0734 for SDG 8 to 0.416 for SDG 10. In particular, we note that SDGs 7, 10, 13, and 17 appear to be rated more positively than the median, while SDG 16 has a more negative sentiment score, as indicated by its scores falling outside the interquartile range of SDGs.

**Fig 4:**
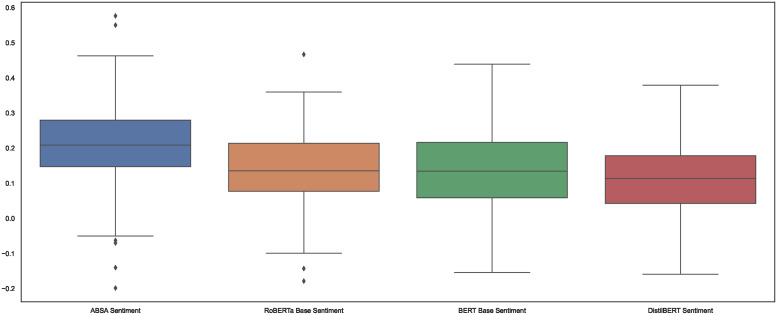
Comparative analysis of sentiment scores: ABSA model vs. vanilla SA models. This figure presents boxplots that compare the overall sentiment scores predicted by our Aspect-Based Sentiment Analysis (ABSA) model with those derived from three baseline vanilla sentiment analysis models—BERT, RoBERTa, and DistilBERT. The boxplots illustrate a slightly higher mean sentiment score for our ABSA model, indicating robust performance. Notably, our ABSA model offers the added benefit of entity-specific sentiment scores, providing a more granular analysis than the general sentiment outputs of the baseline models.

In addition to estimating VNR tone at the entity level, we applied three vanilla SA sentiment models to assess sentiment across all 17 SDGs within the VNRs, and determined the median sentiment score for each. However, we did not train the model; instead, we used the ‘cardiffnlp/twitter-roberta-base-sentiment-latest’, ‘Souvikcmsa/BERT_sentiment_analysis’ and ‘Souvikcmsa/SentimentAnalysisDistillBERT’ models from the transformers pipeline. Fig 4 provides a comparison between the vanilla SA models and our ABSA results, averaged across all 17 SDGs. Overall, the sentiment predictions do not appear to differ qualitatively regarding median sentiment score (ABSA: 0.2088, RoBERTa: 0.1352, BERT-base: 0.1344, DistilBERT: 0.1133) and the standard deviation (ABSA: 0.1086, RoBERTa: 0.1024, BERT-base: 0.1170, DistilBERT: 0.1078). However, the ABSA model offers a more fine-grained analysis.

## 4 Results

### 4.1 Sentiment analysis results

**Fig 5:**
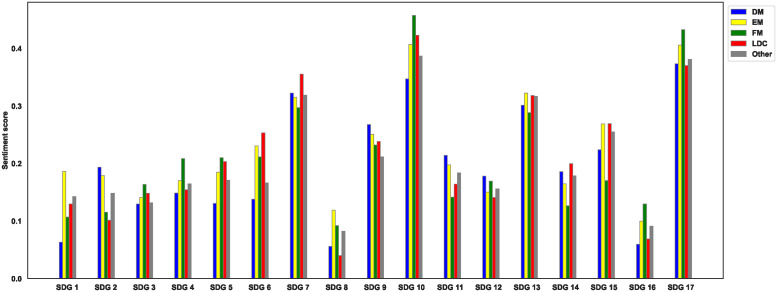
Sentiment score per SDG by MSCI country classification. This figure represents the results of our sentiment analysis for each of the 17 Sustainable Development Goals (SDGs), by country classification. For the 56 countries that published more than one report, we took the average of the countries’ sentiment score, leaving us with 166 countries and their respective scores.

Fig 5 summarizes the sentiment score for each SDG by market classification. We find that LDCs and FM countries tend to report more positively than DM countries. In most cases, either FM countries or LDCs lead in terms of positive reporting on SDGs 3, 4, 5, 6, 7, 10, 14, 15, 16, and 17. Since VNRs are also tools for communicating a country’s progress on each SDG, these findings seem plausible. FM countries and LDCs are generally further away from fully achieving the SDGs, but they appear to be making good progress toward achieving them.

In addition, EM countries report the most positively on SDG 13 ‘climate action’, while the largest differences are found on SDG 1 ‘no poverty’ and SDG 8—’decent work and economic growth’. The latter, in particular, is not surprising, given that emerging economies, such as China and India are experiencing higher economic growth, increased productivity and technological innovation, and, most importantly, a growing middle class [46–48].

Finally, we find that DM countries on average report less positively on most SDGs than EM countries, FM countries, and LDCs do. The exceptions are SDGs 2, 9, 11 and 12, although the differences from second place are negligible. One possible explanation for the results could be that the countries that are already close to achieving some goals are less to report improvements than countries that are at a much lower level of achievement. This possible explanation, according to [49], applies to Australia, where closing the gap to reach 100% of targets seems to be the most difficult. Other DM countries may face the same problems.

### 4.2 Common development paths

Descriptive statistics alone provide only a summary measure and therefore do not describe the spatial (and temporal) variation within VNRs. To explore common development paths and the potential for cross-country collaboration, we used the t-distributed stochastic neighbor embedding (t-SNE) algorithm. This allows us to compress the multidimensional sentiment data related to the 17 SDGs within each VNR into a singular two-dimensional representation suitable for visualization in a scatterplot. This allows us to assess which countries report similarly—positive or negative—about achieving the SDGs and whether other potentially interesting patterns emerge. Clusters of countries on the plot suggest analogous sentiments towards the goals, indicating common challenges or successes related to the 2030 Agenda.

**Fig 6:**
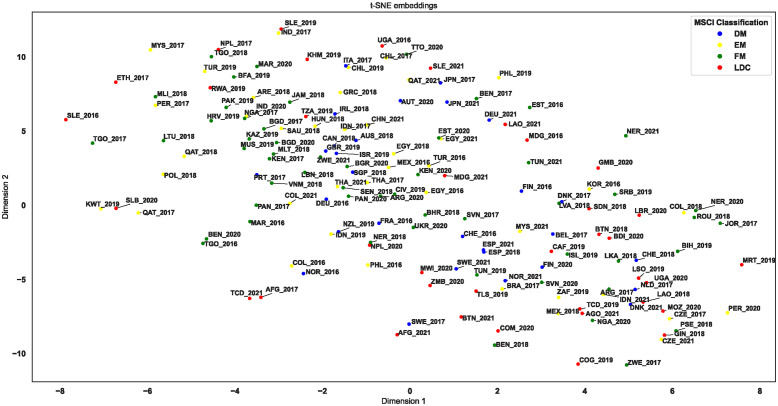
t-SNE embedding of country sentiments towards the 17 SDGs, excluding ‘Others’. This figure illustrates the results of our t-distributed stochastic neighbor embedding (t-SNE) dimensionality reduction by embedding the sentiment towards all 17 Sustainable Development Goals (SDGs) of the countries into a two-dimensional space. The country labels are abbreviated using their ISO3 codes, accompanied by the corresponding publication year. It should be noted that the category ‘Others’ has been intentionally excluded from this figure for illustrative purposes. For the complete representation including the category ‘Others’, please refer to S2 Fig.

Fig 6 provides an overview of all 17 SDGs and 232 VNR reports, which are embedded in a two-dimensional space. Additionally, we report the results of a PCA, a linear dimension reduction technique, that maximizes the variance by preserving large pairwise distances as a robustness check with qualitatively similar results in S1 Fig. One would expect that for countries issuing multiple reports, these documents would cluster closely on the t-SNE plot, reflecting a consistent sentiment score over time. In addition, a narrower time interval between report publications should correlate with proximity on the plot, as minimal changes in sentiment score are expected over shorter periods. This pattern appears to be supported when examining Fig 6.

In general, DM countries appear to be more concentrated in the plot’s center and exhibit a smaller spread compared to EM countries, FM countries, and LDCs. Table 2 presents the statistics for the nearest neighbor distances within each country classification. This analysis reveals that DM countries exhibit the smallest mean and median distances, indicating that the VNRs in this category are generally closer to their nearest neighbors compared to other categories. This suggests a tighter clustering. Furthermore, the standard deviation for DM countries is the lowest, indicating that the distances between points in this category are more consistent than in the other categories.

**Table 2:** Statistics for the nearest neighbor distances within each country classification. This table provides a descriptive analysis of the nearest neighbor distance, which is a measure of proximity between points in a dataset. In the context of our country classifications, these distances quantify the closeness or similarity between countries within the same group, according to the selected criteria.

Country Classification	mean	median	std
DM	0.475	0.473	0.221
EM	0.533	0.531	0.303
FM	0.553	0.529	0.283
LDC	0.591	0.469	0.382
Others	0.632	0.602	0.320

In contrast, we observe greater differences between EM countries, FM countries, LDCs, and the ‘Others,’ where we find considerably greater dispersion (especially among LDCs). Consequently, LDCs exhibit the highest standard deviation. This indicates that they exhibit the most variability in distances to the nearest neighbor with some LDCs being very close and others much further apart. Moreover, the median of the LDCs is considerably lower than its mean, which suggests that the distribution of nearest neighbor distances is skewed, with a greater number of points having a distance less than the mean. The ‘Others’ category has the highest mean distance, indicating that points in this category are, on average, the most distant from their nearest neighbors. This could indicate a greater dispersion within the category or a more heterogeneous composition of points.

Fig 6 also illustrates potential synergies between specific countries. For instance, Canada, Israel, Australia, Great Britain, Ireland, and Singapore have expressed similar sentiments regarding the SDGs in reports spanning from 2018 to 2020. Germany, Austria, and Japan appear to share comparable challenges or achievements in their progress toward the SDGs. Additionally, a cluster is evident for Spain, Switzerland, Finland, Norway, Sweden, and Belgium. Denmark’s reports exhibit greater variation over time. However, in 2017, its report is comparable to those of Finland and Latvia, with the latter two countries’ reports dating to 2016 and 2018, respectively. Further groups can be formed based on the clustering approach, but naming them all would be impractical, particularly given the greater dispersion of FMs, EMs, and LDCs. Nevertheless, in order to identify potential policy implications for a specific country, it is necessary to examine the country in question and the cluster to which it belongs, in order to identify any potential synergies.

In summary, DMs form the most cohesive and uniform cluster, whereas that of LDCs and the ‘Others’ category is more dispersed and variable. The median distances, particularly for LDCs, suggest that the distribution of distances is not symmetrical, and that there may be outliers influencing the mean.

### 4.3 Sentiment scores vs. SDG scores

To compare VNR sentiment scores with actual country performance, we employed Kendall’s rank correlation coefficient as the analytical framework, as illustrated in Table 3. The correlation between the outcomes of our ABSA model and the official UN SDG scores was determined. We opted for Kendall’s Tau instead of Pearson’s correlation coefficient, as the latter assumes a normal distribution of data and a linear relationship between variables, conditions that may not hold true due to the nature of our sentiment dataset. Due to the brevity of some VNRs or the limited scope of their discussion of specific SDGs, it is not uncommon for the sentiment score to be recorded as 1 or -1 at the country level. Furthermore, we selected Kendall’s Tau over Spearman’s correlation coefficient due to its robustness. In order to conduct our analysis, we utilized a cross-data sample that included all countries for which we had access to VNRs. As the scores are derived from a combination of indicators from different years, the mean sentiment score for each country was calculated across all years that the countries reported, ensuring that the period was matched. This procedure yielded a mean SDG sentiment score for each country and an SDG score per SDG. Our hypothesis is that a country underperforming in achieving a SDG will exhibit a low SDG score. This is likely to be reflected in their reports with a negative tone. Consequently, we hypothesize that there is a positive correlation between the sentiment scores and the SDG scores.

**Table 3:** Kendall rank correlation between sentiment score and SDG score. This table displays the outcomes of a Kendall rank correlation analysis that compares the sentiment score derived from our ABSA model for each Sustainable Development Goal (SDG) with the official UN SDG Index scores. The ‘Overall category’ refers to the inclusion of all countries in the analysis, while DM (Developed Markets), EM (Emerging Markets), FM (Frontier Markets), LDC (Least Developed Countries), and ‘Others’ represent the specific country classifications consistently used throughout the paper.

SDG	Overall	DM	EM	FM	LDC	Others
SDG 1	0.004	0.068	0.035	-0.234	0.169	0.086
SDG 2	0.114**	0.176	0.000	0.149	0.236**	0.036
SDG 3	0.005	-0.361***	0.004	-0.160	0.197*	0.046
SDG 4	-0.009	0.110	-0.216*	-0.043	0.112	-0.019
SDG 5	-0.124***	-0.330**	-0.237*	-0.202	0.123	0.007
SDG 6	-0.081*	0.087	-0.149	0.155	-0.111	0.018
SDG 7	-0.010	-0.285**	-0.142	0.080	-0.102	0.071
SDG 8	0.045	-0.142	-0.104	-0.208	0.126	0.099
SDG 9	0.083*	-0.281**	0.132	0.032	0.103	0.105
SDG 10	-0.064	-0.249*	-0.200	0.066	0.105	-0.004
SDG 11	0.102**	-0.303**	-0.236*	0.097	0.192*	0.191**
SDG 12	-0.046	0.016	-0.015	0.096	0.021	-0.033
SDG 13	0.014	0.129	-0.144	0.213	0.154	-0.047
SDG 14	-0.059	-0.122	0.078	-0.146	-0.097	-0.038
SDG 15	-0.035	-0.086	-0.111	0.396**	-0.090	-0.011
SDG 16	0.051	-0.222	-0.033	-0.064	0.313***	0.093
SDG 17	0.002	-0.390***	-0.156	0.144	0.028	0.138*

Note:

*p < 0.1;

**p < 0.05;

***p < 0.01.

Table 3 reveals a relationship that differs from the initial hypothesis, particularly when analyzing the collective data from all countries (Overall). The findings indicate that there is no relationship for the majority of SDGs, as indicated by the lack of significant coefficients at conventional significance levels. Nevertheless, there are exceptions. Notably, SDGs 2 and 11 exhibit significant and positive correlations. This suggests that countries with higher scores on these SDGs tend to report more positive towards them, and vice versa. Conversely, SDG 5 exhibits a significant negative correlation, indicating that countries with lower SDG scores for SDG 5 tend to report more positively on this goal, and vice versa.

Upon examination of the disaggregated correlations by country classification, it becomes evident that the results exhibit a greater degree of distinct patterns. In the case of the DMs, a pronounced negative correlation is observed for SDG 3 and SDG 17, which is significant at the 1% level. This suggests that higher SDG scores on these SDGs may be associated with more negative sentiment score. Additionally, we observed negative correlations for SDGs 5, 7, 9, and 11 at the 5% significance level, and to some extent for SDG 10 at the 1% level, which provides additional support for the notion that greater SDG achievements might correspond with more negative sentiment score within these categories.

For EMs, the correlations are largely non-significant. However, there are exceptions: SDGs 4, 5, and 11 show significant negative correlations at the 10% level. For FMs, SDG 15 is noteworthy for its robust positive correlation, indicating that higher SDG scores are associated with more positive sentiment score. Similarly, LDCs demonstrate positive correlations for SDGs 2, 3, and 11, with SDG 2 exhibiting a particularly robust positive relationship. Furthermore, countries classified as ‘Others’ also exhibit significant positive correlations for SDGs 11 at the 5% and 17 at the 10% level.

These findings, which are disaggregated by country classification, reveal that the relationship between SDG scores and sentiment scores is complex and varies considerably across different country classifications. This highlights the importance of considering these contexts when analyzing sentiment towards SDGs. This is consistent with the results presented in Fig 5, which indicate that countries that are perceived to be less capable of achieving the goals (LDCs and FM countries) tend to write more positively about them in their reports.

## 5 Discussion

Our study makes a distinctive contribution by conducting a comprehensive sentiment analysis of VNRs. In order to explore each country’s perspective on its progress toward the SDGs, we employed the ABSA method. While previous research has shed light on the dynamics of sustainable development, the trade-offs, synergies, and geographical patterns associated with SDGs [4, 7–12, 14, 15], our use of ABSA to analyze VNRs introduces a novel and pivotal angle to the field. In contrast to existing studies that primarily focus on textual similarities [6] or rely on UN-provided indices [4, 7–9, 12, 14, 15], our methodology places emphasis on the sentiment expressed by countries regarding their SDG progress. In this way, we prioritize the self-evaluations of nations, thereby capturing a more complementary view of their challenges and successes than is possible with traditional indices. The t-SNE algorithm has been employed in an analysis of the data, which has yielded insights into the patterns of reporting by countries on their progress towards the SDGs. By reducing the multi-dimensional sentiment data to a two-dimensional space, we have created a visual representation of the similarities and differences in countries’ sentiments towards the SDGs. The observed clustering in the scatter plot suggests that countries with similar sentiment scores may face shared challenges or successes, which could be pivotal for cross-country collaboration within the 2030 Agenda framework. This appears to be particularly the case for DM countries, as they are more centralized in the visualization, indicating a more uniform reporting sentiment, whereas EM, FM and LDCs show a broader dispersion. This could reflect the varying degrees of challenges faced by these countries, with LDCs appear to exhibit the most variability, suggesting a diverse range of experiences and responses to the SDGs. The close grouping of countries, including Switzerland, Sweden, Norway, Denmark, and Finland in the two-dimensional embedding, all of which are among the top 10 most sustainable countries worldwide [50], aligns with findings from Sebestyén et al. [6]. In particular, we observe analogous clusters in the context of reported sentiments, which is consistent with the keyword clustering approach proposed by Sebestyén et al. [6]. For instance, as demonstrated in our clustering approach, Australia is situated in close proximity to Canada and Ireland. Consequently, not only do these countries share similar sentiments, but they also face similar specific challenges in achieving the SDGs. This suggests that they could potentially benefit from exploring synergies and collaborating on strategies to overcome shared obstacles. Additionally, Switzerland, Norway, and Finland are identified as a group in both the results of Sebestyén et al. [6] and our study. While we find some similar groupings to those in Sebestyén et al. [6], there are also differences. For example, Italy, Spain, Montenegro, Portugal, and Greece do not form a close group in our results. This suggests that there are differences between some countries with regard to the topics covered in VNRs regarding the SDGs and the sentiment towards the SDGs’ progress. It would be advantageous for policymakers to establish forums where these exemplary countries can disseminate best practices and insights, with the objective of accelerating progress towards the achievement of the 2030 Agenda.

However, the variability in sentiment scores for countries publishing multiple reports, especially in the context of the COVID-19 pandemic, which brought significant additional challenges to achieving the SDGs by 2030 [51], indicates that external factors such as global crises can significantly influence the narrative of progress. This is particularly evident for approximately half of the countries, for which two or more reports are available before and after the year 2020. The availability of reports from the same country from different periods allows us to conduct a comparative analysis that differs from that of Sebestyén et al. [6] who do not include the time horizon in their analysis. For instance, VNRs from Peru, Nepal, and Argentina in South America; Benin, Chad, Nigeria, Uganda, and Zimbabwe in Africa; as well as Malaysia, Laos, Indonesia, and Qatar in Asia, demonstrate that the sentiment expressed in reporting has undergone a significant transformation over time. This underscores the necessity for policies that are sufficiently flexible and robust to withstand external shocks. In light of this knowledge, policymakers must utilize it to create resilient strategies that ensure continuous progress towards the SDGs, even in the face of unforeseen challenges.

Similarly to Pradhan et al. [9], we employ a correlation analysis. However, we do not examine correlations between SDG scores to identify potential synergies or trade-offs. Instead, our objective is to ascertain whether the SDG scores align with the reporting sentiment of the countries themselves. The Kendall rank correlation coefficient was employed to compare the sentiment expressed in VNRs with the actual SDG performance scores. In contrast to our initial hypothesis, which predicted a positive correlation between VNR sentiment scores and SDG scores, we found an absence of significant correlations for the majority of SDGs on a collective level. Notably, SDGs 2 and 11 show significant positive correlations, while SDG 5 presents a negative one, indicating a complex relationship between reported sentiments and actual performance. A further analysis by country classification revealed some interesting patterns. It can be observed that DM countries often report higher SDG achievements with a critical tone, as indicated by the negative correlations.This could be indicative of the fact that despite their progress, such countries may report with a critical perspective, which may be reflective of higher self-imposed standards or expectations. Conversely, FMs and Least LDCs expressed a more positive tone in general. This positivity may be attributed to the perception that making advancements from a lower starting point seems less daunting than the challenging endeavor of approaching the SDG finishing line for countries that are already on the brink of achieving these goals [49]. In contrast, the data indicates that there are significant negative correlations between the DMs and certain SDGs, while the FMs and LDCs exhibit positive correlations for others, such as SDG 16 and SDGs 2, 3, and 11, respectively. The critical tone observed in the VNRs of DM countries, despite their high SDG scores, suggests that self-assessment can be a powerful tool for continuous improvement. One could argue that policymakers in these countries should maintain a critical perspective to identify areas for further development and avoid complacency.

In summary, the findings indicate that the sentiment expressed in VNRs may not consistently reflect a country’s actual progress on the SDGs. Rather, they appear to be shaped by a variety of factors, including political, economic, and social contexts, as well as the distinct reporting styles and strategies of the countries in question. This complexity suggests that VNRs require a more nuanced interpretation that considers the broader context in which these reports are produced and also indicates the need for differentiated policy approaches. We recommend that policymakers consider the unique contexts of each country classification when formulating SDG-related policies. In particular, for LDCs, it is crucial to develop policies that foster positive sentiment and active engagement with the SDGs, as our findings indicate that positive reporting may not always correlate with high performance. However, the quality of SDG scores is also open to question [16, 17]. Consequently, the results of our study may also be attributed to the lack of SDG score quality. Therefore, it is recommended that policy-makers consider using VNRs as an addition to traditional numerical data in the form of SDG scores.

The findings of our study offer new insights into the field, yet it is essential to acknowledge the limitations of the study and the avenues they open for future research. Firstly, the t-SNE algorithm, while effective for visualization, may not fully capture the multi-dimensional nature of sentiment data. Although we have supplemented this with a PCA dimension reduction, which yields similar results, the full complexity of the data might still be underrepresented. Moreover, the use of Kendall’s Tau, despite its robustness, may not fully capture all the nuances of the relationship between sentiment and SDG scores. Additionally, the issue of outliers due to truncated SDG texts in some reports presented a further challenge. The correlation analysis between sentiment scores and the official UN SDG scores reveals significant variations across different country classifications, further highlighting the inherent complexities in conducting such analyses. The inconsistency in VNR lengths and the selective focus on certain SDGs further complicate a comprehensive analysis. We recommend that the UN to provide more comprehensive guidelines regarding the length and structure of reports. Such measures would not only facilitate a more standardized comparison but also aid in recognizing shared pathways for development across different nations.

One significant challenge was the language barrier, as approximately one-third of VNRs were not published in English and thus required translation using NLP tools. While this approach enabled the inclusion of these reports, it is possible that translations may not fully capture the nuances of sentiment and specific terminology, which could result in the loss of crucial information. As other authors, such as Sebestyén et al. [6], who face the same issue and therefore only had 75 VNRs available in their study, this reinforces our recommendation that VNRs be published in English in addition to the national languages. This will ensure inclusivity and a wider scope for analysis. Such a measure would facilitate research and promote global understanding and collaboration with respect to the SDGs.

The absence of a consistent annual publication requirement for VNRs limited our ability to undertake a detailed time-series analysis from 2016 to 2021. Once more countries have published a second report, a time-series analysis could be a viable option to provide further details on how and whether the tone of VNRs has changed over the years. A comparable issue was encountered when attempting to utilize the official UN scores as indicators, which serves to reinforce the aforementioned commentary on the quality of SDG scores. The aggregated UN score for a single SDG is not published as a time series; rather, it is presented as a combination of several sub-indicators from various years. Previous studies [4, 7–9, 12, 14, 15] might have benefited from a more comprehensive dataset, which would have included a continuous time series. Consequently, future research could concentrate on utilising alternative indicators (e.g., GDP per capita) to facilitate a comparative analysis of sentiment across reports. Future research could, similar to D’Adamo et al. [14], but using NLP techniques, perform an analysis on a regional basis if sufficient data is available, as the model we used is applicable to any text. Therefore, if texts on the progress of the SDGs are available on a regional level, our analysis could also be conducted on a disaggregated level. One disadvantage of employing the sentiment of reports in place of keywords, as exemplified by Sebestyén et al. [6], is that it precludes the formulation of targeted policy recommendations for specific countries with respect to the SDGs. However, our advantage lies in the fact that the sentiment may be able to capture progress, rather than merely identifying similar SDG themes across countries.

The insights from our study also have implications for future research and policy-making. The patterns observed in sentiment reporting could facilitate cross-country collaborations and facilitate the sharing of best practices and the devising of strategies to tackle common challenges. Moreover, the discrepancies between sentiments and actual SDG performance underscore the necessity for a more nuanced approach to the analysis and interpretation of VNRs. Future research should examine the factors that influence the sentiment of reports, including the impact of global events such as the COVID-19 pandemic. An investigation into the reasons behind the tendency of DMs to present a critical outlook despite achieving high scores on the SDGs provide insight into the intricacies of self-assessment within the SDG framework. This investigation could reveal insights into the dynamics of reporting and the actualization of sustainable development goals, thereby providing a more comprehensive understanding for for policy-makers and stakeholders.

## 6 Conclusion

In our analysis, we used an advanced sentiment analysis technique, ABSA, to examine the sentiment expressed in VNRs concerning the SDGs. This is the first comprehensive sentiment analysis of VNRs, offering an in-depth analysis of a country’s distinctive perspective as they report on their progress towards the 2030 Agenda. We trained an ABSA model on UN progress reports and then applied the model to the reviews. Consequently, we generated sentiment score for each country on each of the 17 SDGs. First, we applied t-SNE to our results in order to identify similarities across countries that have published VNRs. Our findings indicate that while there is a consistent sentiment exhibited by DMs over time, there is a notable dispersion in the sentiments of FMs and LDCs. This reflects the varied challenges and developmental stages that these countries face. Secondly, we examined the overall sentiment expressed by the countries in question. Our findings indicated that FM countries and LDCs tend to report more positively than DM countries in their assessments. This phenomenon, which may be described as a ‘progress paradox,’ suggests that initial advancements are often reported with optimism, while the final strides towards full SDG achievement are met with a more critical assessment. One potential explanation for this finding is that achieving an SDG of 100% is typically more challenging than making progress when starting from a lower point [49]. Thirdly, the absence of a significant correlation between the sentiment expressed in VNRs and the actual SDG performance scores for the majority of the SDGs indicates that VNRs may serve more as a reflection of a country’s narrative and outlook rather than as a direct measure of their progress. Consequently, the interpretation of VNRs must be contextualised within the broader framework of each country’s distinctive circumstances and challenges.

Upon returning to our research question, which countries face similar successes and challenges, it can be postulated that a number of countries, including Finland, Switzerland, Spain, Norway, Sweden, and Belgium, among others, appear to be making comparable progress in achieving the SDGs. This suggests that they may benefit from collaboration. Furthermore, the clustering approach can be utilized to identify common development pathways between countries that are closely clustered. The second question, whether SDG scores correlate with reporting sentiments in VNRs, can be answered in the negative. There is no uniform correlation between the sentiment expressed by countries in VNRs and official measures for all SDGs and countries classified according to market.

While our study provides insights into the countries reporting on the progress of the SDGs, there are some drawbacks. Language barriers and translation issues might compromise the accuracy of the data. Furthermore, the variance in sentiment scores across multiple reports, especially when influenced by external shocks such as the COVID-19 pandemic, highlights the sensitivity of narrative sentiment to global crises. In addition, the lack of consistent annual publication of the VNR limits the ability to conduct detailed time-series analyses, which may affect the robustness of conclusions about changes in sentiment over time.

As the 2030 deadline approaches, our research emphasizes the necessity for a nuanced interpretation of VNRs, one that considers the complex interplay of optimism, critical self-assessment, and the tangible realities of SDG implementation. Future research should employ advanced NLP techniques to further explore the sentiment and narratives embedded within VNRs. Furthermore, there is a necessity for the collection of more detailed textual data at the local level in order to facilitate the formulation of nuanced policy recommendations for collaboration. Another potential future research aim could be to understand why there are discrepancies between sentiment reported in VNRs and actual performance on the SDGs, especially in developed markets, where critical tones might indicate higher self-imposed standards or a deeper self-assessment process. Such investigations will not only enhance our comprehension of global SDG advancement but also facilitate more efficacious policy-making and international collaboration towards a sustainable future for all.

## Supporting information

S1 Fig PCA linear dimensionality reduction. This figure illustrates the results of a Principal Component Analysis (PCA), a linear dimensionality reduction technique that maximizes variance and preserves large pairwise distances, by projecting the sentiment towards all 17 Sustainable Development Goals (SDGs) of the countries into a two-dimensional space. The labels for countries are abbreviated using their ISO3 codes, accompanied by the corresponding publication year.
(EPS)

S2 Fig t-SNE embeddings of country sentiments towards the 17 SDGs, including the category ‘Others’. This figure illustrates the results of our t-distributed stochastic neighbor embedding (t-SNE) dimensionality reduction by embedding the sentiment towards all 17 Sustainable Development Goals (SDGs) of the countries into a two-dimensional space. The labels for countries are abbreviated using their ISO3 codes, accompanied by the corresponding publication year. This figure provides a comprehensive view, incorporating the category ‘Others’ to present the full spectrum of data.
(EPS)

S1 Table Cleaning steps of parsed raw texts. This tables presents the various cleaning steps completed to extract text from parsed PDF documents.
(PDF)

S2 Table Analyzed Voluntary National Reviews. This table offers a detailed overview of all the Voluntary National Reviews included in this analysis, detailing their ISO3 country codes, the number of pages, and the year of publication.
(PDF)
