# Supplementary material for: Finding common development paths in voluntary national reviews reporting on sustainable development goals using aspect-based sentiment analysis
Source: PLoS One. 2024 Aug 14;19(8):e0307886. doi: 10.1371/journal.pone.0307886 (PMC11324109; doi:10.1371/journal.pone.0307886)
Supplement: S1 Table — This tables presents the various cleaning steps completed to extract text from parsed PDF documents. (PDF) [file pone.0307886.s003.pdf]

S1 Table. Cleaning steps of parsed raw texts.

Problem	Fix
Extra whitespaces	Replace extra whitespaces with a single whitespace
Words separated by hyphen	Remove hyphen
Words separated by whitespace	Remove whitespace
Model parsed some ff, fi and if characters as one special character	Replace special double characters with normal characters
Whitespaces between word in sentence and punctuation	Remove whitespace
URLs in text which do not have any semantic meaning	Remove all URLs from text
Parsed some sentences character by character with whitespace between them, i.e., S E N T E N C E instead of Sentence. Problem mostly occurred with figure subtexts	As most figure subtexts do not have important semantic meaning, we removed such single characters
Very short sentences or single tokens	Filter out sentences with less than 6 words or 50 characters
Coding errors	Filter out sentences with a high percentage of unrecognized words using a spell checker
