# Supplementary material for: Finding common development paths in voluntary national reviews reporting on sustainable development goals using aspect-based sentiment analysis
Source: PLoS One. 2024 Aug 14;19(8):e0307886. doi: 10.1371/journal.pone.0307886 (PMC11324109; doi:10.1371/journal.pone.0307886)
Supplement: S2 Table — This table offers a detailed overview of all the Voluntary National Reviews included in this analysis, detailing their ISO3 country codes, the number of pages, and the year of publication. (PDF) [file pone.0307886.s004.pdf]

S2 Table. Analyzed Voluntary National Reviews.

Country	Abb.	Pages	Year	Country	Abb.	Pages	Year
Afghanistan	AFG	81	2017	Afghanistan	AFG	54	2021
Albania	ALB	108	2018	Algeria	DZA	176	2018
Andorra	AND	116	2018	Angola	AGO	237	2021
Antigua and Barbuda	ATG	144	2021	Argentina	ARG	130	2017
Argentina	ARG	252	2020	Armenia	ARM	82	2018
Armenia	ARM	60	2020	Australia	AUS	132	2018
Austria	AUT	116	2020	Azerbaijan	AZE	138	2021
Azerbaijan	AZE	70	2017	Azerbaijan	AZE	137	2019
Bahamas	BHS	159	2018	Bahrain	BHR	106	2018
Bangladesh	BGD	82	2017	Bangladesh	BGD	201	2020
Belgium	BEL	95	2017	Belize	BLZ	51	2017
Benin	BEN	80	2017	Benin	BEN	92	2018
Benin	BEN	94	2020	Bhutan	BTN	116	2021
Bhutan	BTN	86	2018	Bolivia	BOL	115	2021
Bosnia and Herzegovina	BIH	96	2019	Brazil	BRA	41	2017
Brunei Darussalam	BRN	113	2020	Bulgaria	BGR	132	2020
Burkina Faso	BFA	117	2019	Burundi	BDI	138	2020
Cabo Verde	CPV	86	2018	Cabo Verde	CPV	168	2021
Cambodia	KHM	116	2019	Cameroon	CMR	200	2019
Canada	CAN	148	2018	Central African Republic	CAF	120	2019
Chad	TCD	116	2019	Chad	TCD	100	2021
Chile	CHL	258	2019	Chile	CHL	133	2017
China	CHN	84	2021	Colombia	COL	74	2016
Colombia	COL	136	2018	Colombia	COL	166	2021
Comoros	COM	112	2020	Congo	COG	119	2019
Democratic Republic of the Congo	COD	124	2020	Costa Rica	CRI	119	2017
Costa Rica	CRI	156	2020	Croatia	HRV	109	2019
Cuba	CUB	124	2021	Cyprus	CYP	148	2021
Cyprus	CYP	81	2017	Czech Republic	CZE	82	2021
Czech Republic	CZE	40	2017	Cote divoire	CIV	153	2019
Denmark	DNK	140	2017	Denmark	DNK	294	2021
Dominican Republic	DOM	274	2018	Dominican Republic	DOM	124	2021
Ecuador	ECU	202	2018	Ecuador	ECU	238	2020
Egypt	EGY	59	2016	Egypt	EGY	72	2018
Egypt	EGY	92	2021	El Salvador	SLV	50	2017
Estonia	EST	60	2016	Estonia	EST	104	2020
Eswatini	SWZ	79	2019	Ethiopia	ETH	52	2017
Fiji	FJI	108	2019	Finland	FIN	64	2016
Finland	FIN	172	2020	France	FRA	53	2016
Gambia	GMB	191	2020	Georgia	GEO	16	2016
Georgia	GEO	69	2020	Germany	DEU	144	2021
Germany	DEU	59	2016	Ghana	GHA	118	2019
Greece	GRC	160	2018	Guatemala	GTM	277	2017
Guatemala	GTM	459	2019	Guinea	GIN	122	2018
Guyana	GUY	178	2019	Honduras	HND	52	2017
Honduras	HND	108	2020	Hungary	HUN	85	2018
Iceland	ISL	151	2019	India	IND	41	2017
India	IND	188	2020	Indonesia	IDN	786	2021
Indonesia	IDN	298	2019	Indonesia	IDN	138	2017
Iraq	IRQ	95	2019	Iraq	IRQ	123	2021
Ireland	IRL	300	2018	Israel	ISR	430	2019
Italy	ITA	50	2017	Jamaica	JAM	163	2018
Japan	JPN	258	2021	Japan	JPN	52	2017
Jordan	JOR	70	2017	Kazakhstan	KAZ	159	2019
Kenya	KEN	124	2020	Kenya	KEN	76	2017
Democratic Peoples Republic of Korea	PRK	66	2021	Republic of Korea	KOR	34	2016
Kuwait	KWT	115	2019	Kyrgyzstan Republic	KGZ	182	2020
Lao PDR	LAO	130	2018	Lao PDR	LAO	156	2021
Latvia	LVA	63	2018	Lebanon	LBN	94	2018
Lesotho	LSO	145	2019	Liberia	LBR	147	2020
Liechtenstein	LIE	77	2019	Lithuania	LTU	70	2018
Luxembourg	LUX	46	2017	Madagascar	MDG	42	2016
Madagascar	MDG	86	2021	Malawi	MWI	104	2020
Malaysia	MYS	82	2017	Malaysia	MYS	144	2021
Maldives	MDV	28	2017	Mali	MLI	70	2018
Malta	MLT	122	2018	Marshall Islands	MHL	147	2021

S3 Table continued on next page

Country	Abb.	Pages	Year	Country	Abb.	Pages	Year
Mauritania	MRT	96	2019	Mauritius	MUS	140	2019
Mexico	MEX	112	2016	Mexico	MEX	162	2018
Micronesia	FSM	140	2020	Moldova	MDA	171	2020
Monaco	MCO	70	2017	Mongolia	MNG	97	2019
Montenegro	MNE	163	2016	Morocco	MAR	11	2016
Morocco	MAR	214	2020	Mozambique	MOZ	68	2020
Namibia	NAM	82	2021	Namibia	NAM	44	2018
Nepal	NPL	52	2017	Nepal	NPL	104	2020
Netherlands	NLD	44	2017	New Zealand	NZL	63	2019
Nicaragua	NIC	105	2021	Niger	NER	75	2018
Niger	NER	131	2020	Niger	NER	85	2021
Nigeria	NGA	100	2017	Nigeria	NGA	120	2020
North Macedonia	MKD	148	2020	Norway	NOR	29	2016
Norway	NOR	124	2021	Pakistan	PAK	81	2019
Palau	PLW	107	2019	State of Palestine	PSE	128	2018
Panama	PAN	106	2017	Panama	PAN	386	2020
Papua New Guinea	PNG	63	2020	Paraguay	PRY	100	2018
Paraguay	PRY	432	2021	Peru	PER	67	2017
Peru	PER	146	2020	Philippines	PHL	27	2016
Philippines	PHL	50	2019	Poland	POL	106	2018
Portugal	PRT	89	2017	Qatar	QAT	184	2021
Qatar	QAT	44	2018	Qatar	QAT	52	2017
Romania	ROU	94	2018	Russian Federation	RUS	228	2020
Rwanda	RWA	124	2019	Saint Lucia	LCA	51	2019
Samoa	WSM	87	2020	San Marino	SMR	109	2021
Saudi Arabia	SAU	96	2018	Senegal	SEN	153	2018
Serbia	SRB	103	2019	Seychelles	SYC	132	2020
Sierra Leone	SLE	81	2021	Sierra Leone	SLE	56	2016
Sierra Leone	SLE	54	2019	Singapore	SGP	84	2018
Slovakia	SVK	33	2018	Slovenia	SVN	78	2017
Slovenia	SVN	100	2020	Solomon Islands	SLB	106	2020
South Africa	ZAF	130	2019	Spain	ESP	181	2018
Spain	ESP	352	2021	Sri Lanka	LKA	115	2018
Sudan	SDN	62	2018	Sweden	SWE	148	2021
Sweden	SWE	88	2017	Switzerland	CHE	28	2018
Switzerland	CHE	28	2016	Tajikistan	TJK	46	2017
Tanzania	TZA	186	2019	Thailand	THA	94	2017
Thailand	THA	83	2021	Timor Leste	TLS	198	2019
Togo	TGO	32	2016	Togo	TGO	44	2017
Togo	TGO	36	2018	Tonga	TON	84	2019
Trinidad and Tobago	TTO	100	2020	Tunisia	TUN	148	2019
Tunisia	TUN	254	2021	Turkey	TUR	149	2019
Turkey	TUR	50	2016	Turkmenistan	TKM	79	2019
Uganda	UGA	118	2016	Uganda	UGA	104	2020
Ukraine	UKR	117	2020	UAE	ARE	73	2018
UK	GBR	235	2019	Uruguay	URY	386	2017
Uruguay	URY	246	2018	Uruguay	URY	110	2021
Vanuatu	VUT	97	2019	Venezuela	VEN	285	2016
Viet Nam	VNM	94	2018	Zambia	ZMB	112	2020
Zimbabwe	ZWE	58	2017	Zimbabwe	ZWE	144	2021
